# Microglia guide stepwise retinal vascular sprouting and remodeling in postnatal CX3CR1-GFP mice: a spatiotemporal observation from P1 to P30

**DOI:** 10.3389/fphys.2026.1926267

**Published:** 2026-10-28

**Authors:** Jiaojiao Wang, Qian Liu, Pingling Shi, Yange Wang

**Affiliations:** Henan Provincial People’s Hospital, Henan Eye Hospital, People’s Hospital of Zhengzhou University, Zhengzhou, Henan, China

**Keywords:** CX3CR1 gfp/gfp mice, development, etina, microglia, vascular

## Abstract

**Introduction:**

Retinal vessel formation is a complex process that may be affected by multiple etiological factors. This study aimed to investigate the formation of retinal vessels in CX3CR1 gfp/gfp mice after birth.

**Methods:**

CX3CR1 gfp/gfp transgenic mice were subjected to retinal vascular examination at eight different time points during development. The differentiation of arteries and veins, branching of vessels, and vessel length were thoroughly examined. The potential correlation between developmental vessels and microglia were examined during the developmental process.

**Results:**

We found that retinal vessels developed from the center of the optic papilla and radiated to the peripheral retina. Vascular remodeling began at P7. Subsequently, at P14, the vascular network consolidated with a honeycomb-like architecture. The length of the large vessels increased gradually from P14 until a complete vascular network was formed at P17. The number of primary branches increased from P17 and then decreased until P30. The density of microglia in the central retina was higher than at the edge retina between P1 and P30. We found that microglia appeared at the edge of the retina and preferred vessel formation at P1. Retinal microglia are involved in retinal vessel formation and development.

**Discussion:**

These findings may serve as a reference database for therapeutic trials on retinal vascular diseases.

## Introduction

Vasculature forms a highly complicated structure of interconnected vessels providing elementary nutrients, growth factors, and oxygen for the developing tissue (Carmeliet and Jain, 2011; Waheed et al., 2015) and developmental malformations or pathological changes in the vasculature may result in fatal consequences, especially for organs with high energy demands, such as the retina (Eelen et al., 2020). Aberrant growth of neovessels in the retina causes visual impairment in common blinding disorders, including retinopathy of prematurity, proliferative diabetic retinopathy, and age-related macular degeneration (Villacampa et al., 2020).

Various animal models have been developed to study the underlying mechanisms of these sight-threatening conditions. Among the numerous animal species used in these models, mouse is a well-established platform for creating systemic or conditional transgenic strains (Stahl et al., 2010). In the mouse retina, the outermost layer, including the photoreceptor layer, is nourished by the choroidal vasculature. The inner retinal vasculature supports the superficial layers of the retina. Retinal vasculature emerges from the central retinal artery and gives rise to interconnected vascular networks (Alves et al., 2020). Therefore, a detailed evaluation of layer-specific vascular development is vital for studying the pathological processes of retinal diseases.

Microglia are specialized tissue macrophages of the central nervous system (Grassivaro et al., 2020; Haage et al., 2019) and rank as the third type of glial cells in the retina. Microglia are involved in a series of physiological activities such as immune regulation (Mbagwu et al., 2019; Xu et al., 2020), tissue development (Kracht et al., 2020), self-stabilization (Tabor et al., 2021) and wound repair (Mesquida-Veny et al., 2021). Microglia are closely correlated with the architecture and function of vascular networks. Studies have shown that microglia migrate to tissues and vessels during retinal development. They may act as the ‘vessel-associated’ microglial populations and include cells bearing macrophage markers (Provis, 2001). A recent study showed that a decrease in the number of retinal microglial cells is accompanied by reduced retinal vessel density (Checchin et al., 2006). Researchers injected microglia into the vitreous bodies of neonatal mice and found that they alleviated the decrease in retinal vascular density during development. These findings highlight that microglia have specific effects on retinal vascular development (Ebneter et al., 2017). However, the exact relationship between microglia and vascular development remains unclear. The interactions between microglia and blood vessel development need to be thoroughly characterized.

Laser microscopy and 3D imaging technology provide novel tools for studying vascular architecture in mice. CX3CR1(gfp/gfp) mice are an excellent model for studying the developmental process of microglia (Zhang et al., 2013). In this transgenic mouse, the CX3CR1 gene was replaced with the green fluorescent protein gfp DNA sequence [CX3CR1(gfp/gfp)]. In contrast, *in vivo* imaging technologies facilitate the visualization of retinal cells within the retina. These advantages collectively allow longitudinal microglia assessment in neonatal mice (Rust et al., 2019). We used laser microscopy and 3D imaging technology to study retinal vasculature development in CX3CR1 GFP/GFP transgenic mice. The interactions between retinal microglia and vascular development have also been characterized. Here, we present an exact timeline of retinal vascular development. Differentiation patterns, branching mode, and vascular length were examined at different time points. We found that a complete vascular network was formed at P17, and vascular differentiation started at P14. The density of microglia in the central retina was higher than that at the edge retina at P1, P10, and P30. However, the differences were not statistically significant. Moreover, microglia appeared at the edge of the retina in preference to vessels at P1. These findings may serve as a reference database for therapeutic experiments on retinal vascular diseases.

## Methods

### Animals and experimental design

The animals were handled according to the ARVO guidelines for using animals in ophthalmic and vision research. All experimental protocols were approved by the Experimental Animal Ethics Committee of Henan Eye Institute(HNEECA-2017-19). A total of 68 CX3CR1 (gfp/gfp) mice (purchased from the Animal Center of Wenzhou Medical University, China) were used in this study. CX3CR1 (gfp/gfp) mice express the green fluorescent protein gfp under the promoter of CX3CR1 (C57BL/6-CX3CR1) and can be used to image microglial cells *in vivo* (Potente et al., 2011). Five female mice (born on March 21, 2019) and five male mice (born on March 31, 2019) were mated and bred together. Animals were housed in a specific pathogen-free (SPF) facility (18–23 °C, 40–65% humidity) with food and water available ad libitum. Female mice were given eggs and seeds in addition to normal food from pregnancy to childbirth. These mice were examined between P1 and P30 (P1, P4, P7, P10, P14, P17, P20, and P30, respectively). Six mice were selected for evaluation at each time point. The mice were sacrificed, and their retinas were prepared as retinal wholemounts. The vessels and microglia in the retinal wholemount were examined using fluorescence microscopy and laser confocal scanning microscopy. The examination items included the length of large vessels, the number of primary branches of the vessels, the density of microglia in the center and edge retina, and the percentage of blood vessel area in the center and edge retina. In addition, minocycline (33 mg/kg) was intraperitoneally administered once daily from the newborn to the end stage to observe microglia and vessels.

### Preparation of retinal wholemount

The mice were anesthetized and killed, and their eyes were enucleated using curved forceps. The eyeballs were immediately placed in 4% paraformaldehyde and fixed at 4 °C for 2 h. The eyeballs were then washed with phosphate-buffered saline (PBS). A small cut was made along the edge of the corneal limbus with a surgical blade, and the cornea, iris, lens, and vitreous were cut off with ophthalmic micro-scissors. The sclera and choroid were gently peeled off, leaving an entire retina intact. The tissue was incubated with Triton-X100 (0.5%, Sigma) and isolectin B4 (1:200, Sigma) overnight at 4 °C. The next day, the tissue was washed with PBS three times for 5 min each. Finally, the retina was cut into four petals toward the optic nerve with a surgical blade and then spread flat on a glass slide. Finally, a coverslip was placed over the entire mount.

### Fluorescence microscope examination

A fluorescence microscope (80i, Nikon, Japan) was used to photograph the retinal region. The whole retina was divided into top, bottom, left, right, and center regions. We took at least five photographs of each region to ensure a global picture of the retina could be assembled. We chose the same field of view on the retina to take photos with a 4x, 10x, and 20x lens, respectively (80i, Nikon, Japan).

### Laser scanning confocal microscope observation

Retina samples were scanned at multiple levels using a laser confocal scanning microscope. The 3D images were captured using a Zeiss LSM 780 NLO fluorescence microscope system (Carl Zeiss Micro-Imaging GmbH, Jena, Germany) and Zeiss control software (Zen 2010; Carl Zeiss Micro-Imaging GmbH) (Yagasaki, et al., 2014). All images were captured using a 20x objective lens. The samples were scanned using a 2-μm z-axis step size to generate 3D datasets from the lowest layer to the upper layer containing microglia and blood vessels in the retina. The image sequences were recorded as 1024×1024-pixel images, and 3D data were collected from the center of the retina.

### Analysis of microglia and vessels

ImageJ software (National Institutes of Health, Bethesda, Maryland, Unites States) was used to analyze the image. The Adaptive-Threshold plug-in was used for threshold processing to produce binary images. We selected the retinal region, and the software automatically counted the number of microglia within the area (Al-Latayfeh et al., 2012). The length of large vessels and the number of primary branches of the vessels were measured using ImageJ and GraphPad software (GraphPad Software, Inc., California, United States). Based on the pixels of the image, 1.7 µm was measured as the minimum length of a pixel. Accordingly, a distance of 0–1.7 µm indicated that microglia and blood vessels were closely connected or that microglia “grew” on the blood vessels, while a distance greater than 1.7 µm indicated that microglia were far away from blood vessels. The percentage of microglia away from blood vessels was quantified using StrataQuest software (version 7.0.1, Tissue Gnostics GmbH, Vienna, Austria).

### Statistical analysis

Continuous data with normal distribution were compared using the paired sample t-test. Otherwise, the Wilcoxon test was used. All data are presented as mean ± standard deviation (SD). P value was set at 0.05.

## Results

### The development process of vessels in the retina

Retinal wholemounts were examined using a fluorescence microscope. We found that the vessels in the retina originated from the center of the visual papilla and grew radially to the periphery; these vessels were sparse and inhomogeneous and did not reach the edge of the retina at P1 (**Figure 1A**). At P4, the vessels spread to the edges and gradually occupied the entire retina, forming a radial and uniform network. However, the trunks and branches of the vessels were not differentiated by P4, and arteries, veins, and their branches could not be distinguished by morphology (**Figure 1B**). At P7, the main vessel was remodeled, and several branches sprouted from the main vessels. (**Figure 1C**). At P10, the vascular network continued to spread, forming a denser network structure at the periphery (**Figure 1D**). At P14, the vascular network in the periphery appeared as a relatively uniform and dense honeycomb-like structure; in the central area, they became relatively loose. Moreover, the arteries and veins could be distinguished by the vessel diameter; the diameter of the vein was much larger than that of the artery (**Figure 1E**). At P17, the minute vessels in the retina were further remodeled; the extra minute vessels disappeared, and these large vessels reached the edge of the retina. The vascular network became sparse, and the vessels had more branch vessels. At this time, a complete vascular network was formed, and the architecture was similar to that of adult mice (**Figure 1F**). At P20 and P30, a loose vascular network was found in the retinas, and the arteries and veins were readily identified by their vessel diameters. (**Figures 1G, H**). Our results showed that retinal vessels radiated from the center of the optic papilla to the peripheral region during development. Vascular remodeling began at P7 and ended at P17.

**Figure 1:**
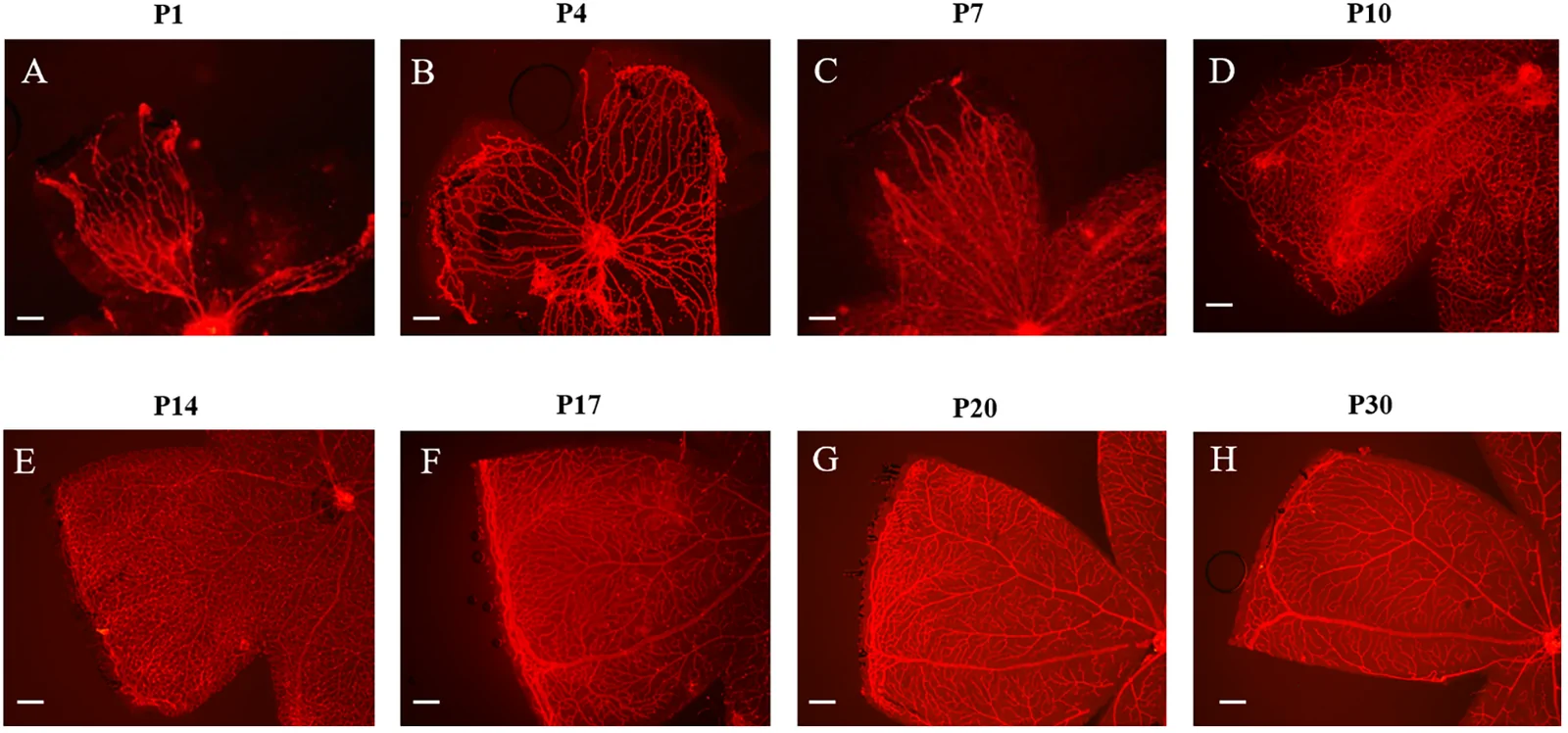
The development process of vessels in the retina. **(A)** The vessels in the retina started from the center of the visual papilla and grew radially to the periphery. The vessels were sparse and non-homogeneous and did not reach the edge of the retina at P1. **(B)** At P4, the vessels continued to spread in the retina with the optic papilla as the center, forming a radial and relatively uniform network structure, gradually occupying the entire retina. During this period, the trunks and branches of vessels had not differentiated, and arteries, veins, and their branches could not be distinguished. **(C)** At P7, vascular remodeling around the main vessel was observed, and the main vessel and branches appeared; however, the arteries and veins were still indistinguishable. **(D)** At P10, the vascular network continued to spread to the periphery of the retina and formed a denser network structure. **(E)** At P14, the vascular network in the periphery presented a relatively uniform and dense honeycomb-like structure, which in the central area became relatively loose. Furthermore, there was a clear difference in blood vessel diameter, and arteries and veins were distinguishable. **(F)** At P17, the minute vessels in the retina were further remodeled, the extra minute vessels disappeared, and the large vessels reached the edge of the retina. The vascular network became sparser, with more branching and grading of vessels. At this point, a complete vascular network had formed, which was not considerably different from that of adult mice. **(G, H)** The vascular network was loose, and the arteries and veins were distinguishable at P20 and P30. (Scale bar: 100 μm).

### Differentiation of arteries and veins in the retinas.

Images from the fluorescence microscope were enlarged to obtain details regarding the differentiation of the arteries and veins. The trunks and branches of the retinal vessels did not differentiate at P4 (**Figure 2A**). At P7, vascular remodeling was observed in the central retina, and the primary branches started to differentiate (**Figure 2B**). At P10, large and small vessels could be distinguished by the diameter of the vessel (**Figure 2C**). By P14, arteries and veins were profoundly differentiated. As the picture shows, the artery was labeled with “a,” and the vein was labeled with “v” (**Figure 2D**). At P17, the large vessels touched the edge of the retina, and the architecture of the vascular network was similar to that of adult mice (**Figure 2E**).

**Figure 2:**
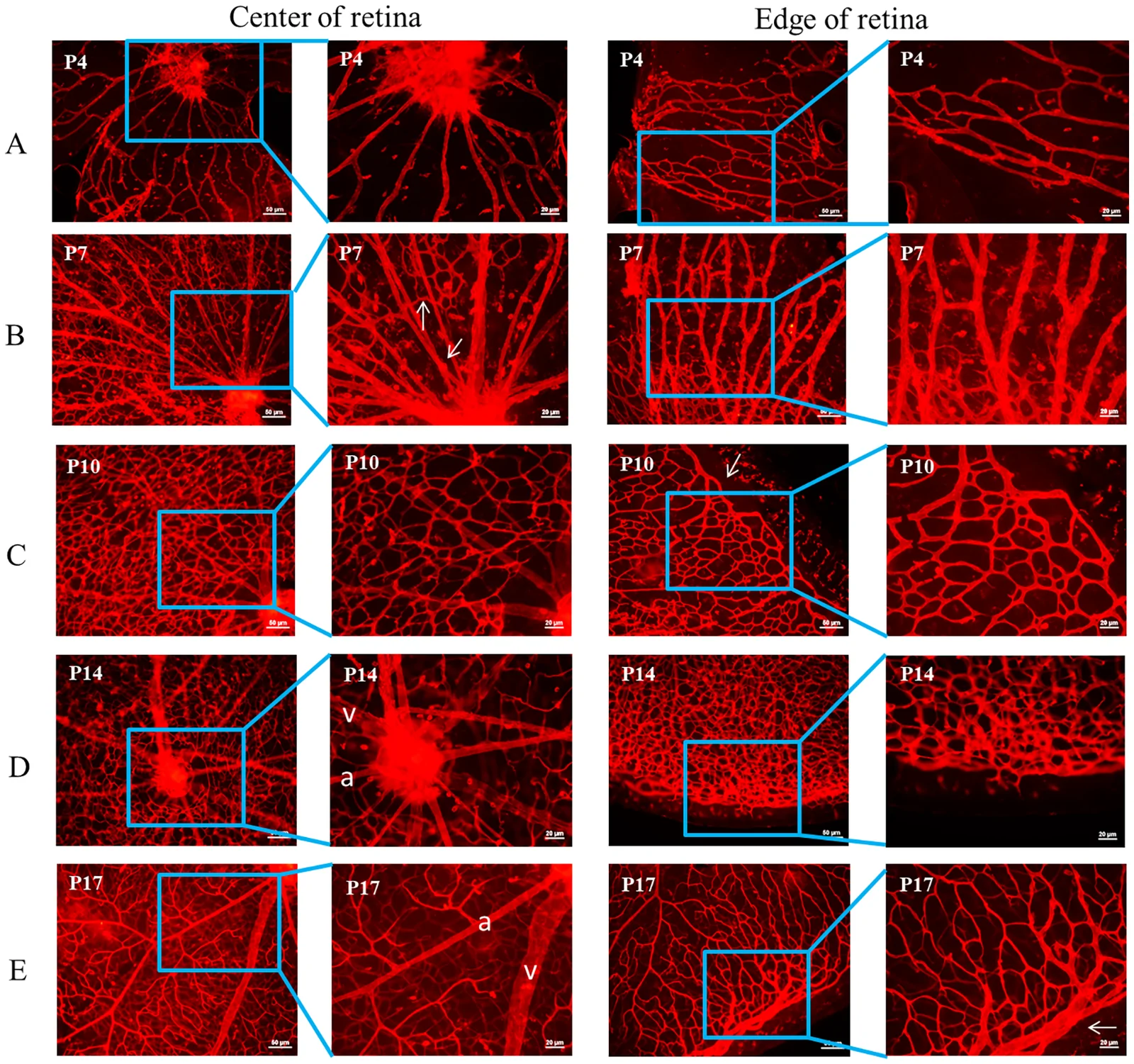
Differentiation of arteries and veins in the retina. A fluorescence microscope with 10x objective and 20x objective was used to image the center and edge of the retina, respectively. **(A)** The trunks and branches of the vessels in the retina were not differentiated, and the arteries and veins could not be distinguished at P4. **(B)** At P7, vascular remodeling was observed around the main vessels in the central retina, and the main branches were differentiated; however, the arteries and veins could not be distinguished. **(C)** At P10, the large and small vessels were more clearly distinguishable, and the small blood vessel network reached the edge of the retina. **(D)** As the retina developed, arteries and veins differentiated at P14. As the picture shows, the artery is labeled “a,” and the vein is labeled “v.” **(E)** At P17, arteries, and veins were more clearly distinguishable in the central retina, which was similar to that in adult mice. It is more important that large vessels touch the edge of the retina. (The blue boxes represent photos taken under a 10x lens. Scale bar, 50 µm. The pictures without blue boxes represent photos taken under a 20x lens, which are enlarged views of blue boxes. Scale bar, 20 µm.).

### Quantitative analysis of retinal vascular development.

To obtain quantitative results of vascular development, we measured the length of large vessels and the number of primary branches of the vessels using ImageJ software. The same examiner marked and manually counted the vessel branches (**Figure 3A**). From P14 to P17, the number of primary branches increased significantly (23.75 ± 1.532 vs. 30.875 ± 1.274; p<0.05; n=8). The length of the large vessels increased from P14 to P17 (2957.784 ± 59.077 vs. 3651.441 ± 115.737); however, the difference was not significant (p>0.05; n=8). At P20, the length of the large vessels increased significantly compared to that at P14 (5279.619 ± 127.833 vs. 2957.784 ± 59.077; p<0.05; n=8). The number of primary branches was also significantly larger than that at P14 (41.25 ± 1.306 vs. 23.75 ± 1.532; p<0.05; n=8). At P30, the length of large vessels increased over time (6281.784 ± 161.789, p<0.05; n=8). In contrast, the number of primary branches decreased (24.375 ± 0.625; n=8) (**Figure 3B**).

**Figure 3:**
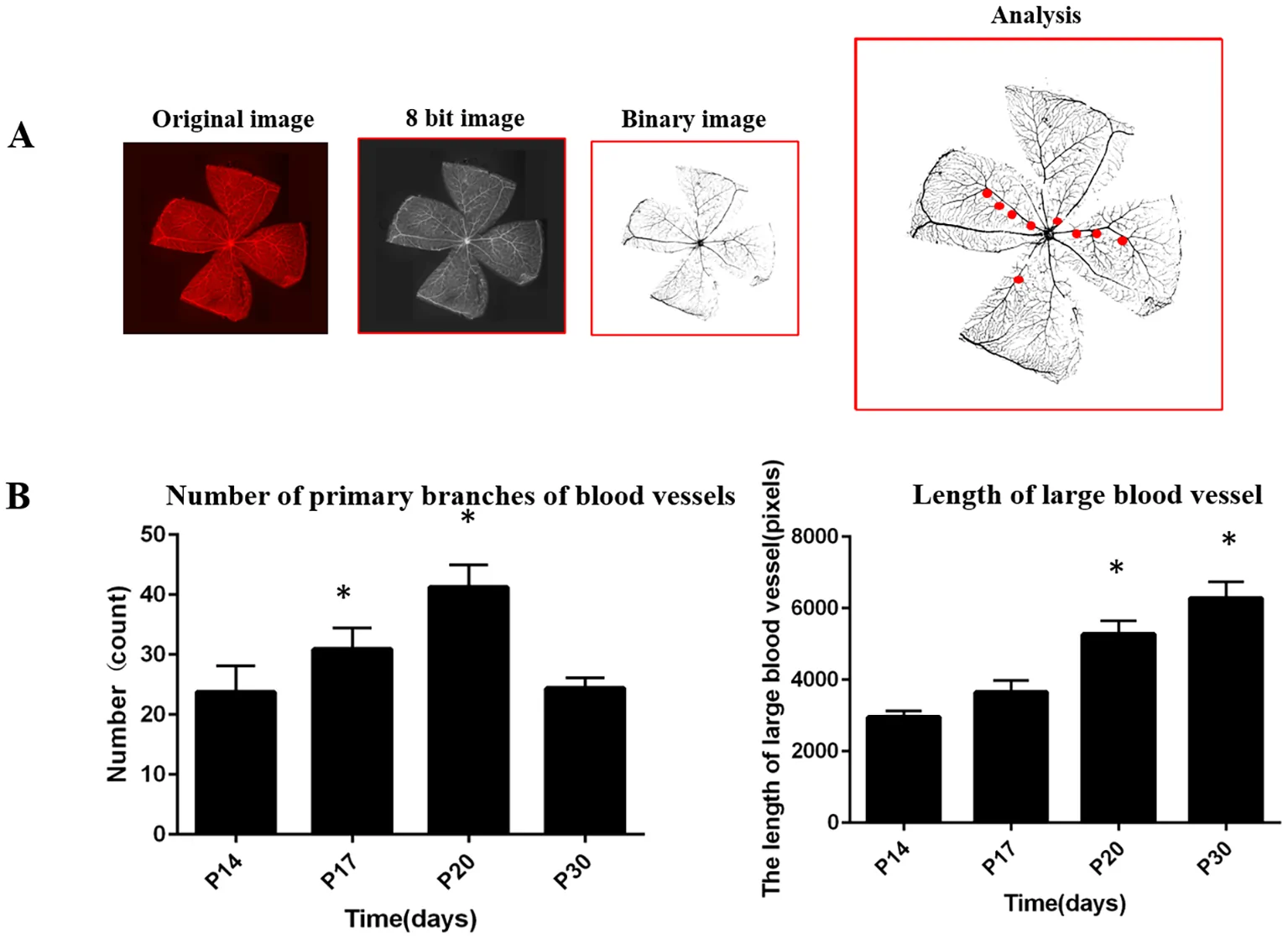
Quantitative analysis of retinal vascular development. **(A)** Vessel branches were manually marked and counted. **(B)** The number of primary branches of vessels increased from P14 to P17, and the difference was significant (*p* < 0.05; n=8). At P20, the number continued to increase. The length of large vessels increased from P14 to P17. However, the difference was not significant (*p*>0.05; n=8). Furthermore, it increased from P20 to P30. These two groups were compared with P14, respectively. The differences were statistically significant (*p* < 0.05; n=8) (Statistical differences were processed using Wilcoxon signed-rank test, with a p value of <0.05 for differences between groups). Asterisks indicate p<0.05.

### Microglia and vessels

Retinal wholemounts were subjected to laser scanning using a confocal microscope. At P1, microglia and vessels were detected in the center region of the retina wholemounts (**Figures 4A–D**). Microglia were found in the peripheral region of the retina, while vessels were not be detected (**Figures 4E–H**). The density of microglia in the central retina was higher than that in the edge retina (7381.31 ± 762.45 cells/mm^2^ vs. 4830.39 ± 352.51 cells/mm^2^); however, the difference was not significant (p>0.05; n=8) (**Figure 4I**). The percentage of vessel area in the central retina was higher than that in the edge retina (4.43 ± 1.24% vs. 3.56 ± 0.37%); however, the difference was not significant (p>0.05; n=8) (**Figure 4J**).

**Figure 4:**
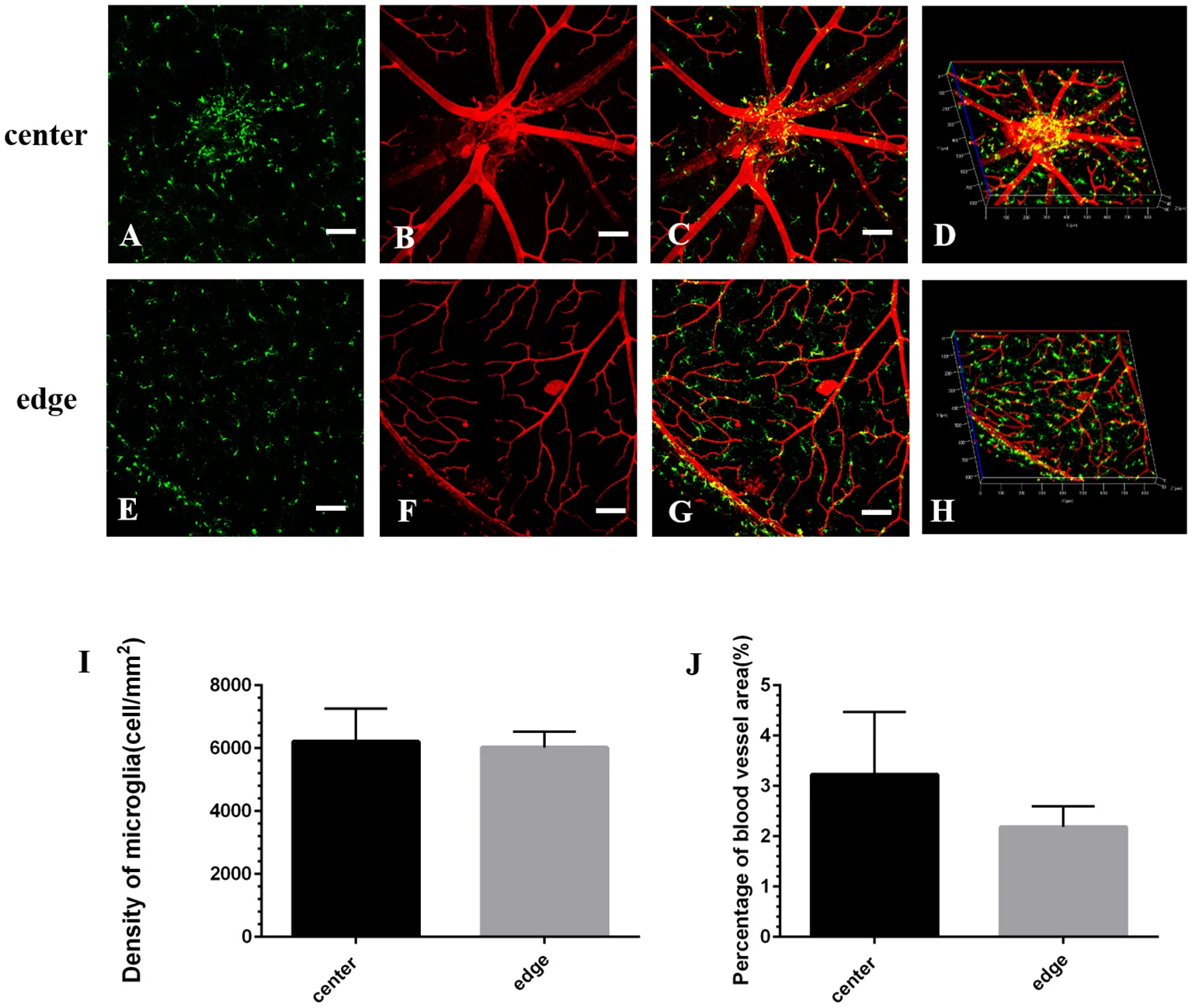
Microglia and vessels at P1. **(A–D)** Microglia and vessels could both be observed in the central retina at P1. **(E–H)** Microglia could be observed in the most peripheral part of the retina; however, vessels had not developed. **(I)** The density of microglia in the center and edge retina. **(J)** The percentage of blood vessel area in the center and edge retina. (Statistical differences were processed with the Wilcoxon signed-rank test with a p value of <0.05 for differences between groups).

At P10, microglia and vessels were found in both the central and peripheral retina (**Figures 5A–H**). The density of microglia in the central retina was higher than that in the edge retina (8405.97 ± 425.38 cells/mm^2^ vs. 6335.71 ± 608.76 cells/mm^2^); however, the difference was not significant (p>0.05; n=8) (**Figure 5I**). The percentage of blood vessel area in the central retina was higher than that in the edge retina (4.66 ± 0.43% vs. 2.87 ± 0.38%), and the difference was significant (p<0.05; n=8) (**Figure 5J**).

**Figure 5:**
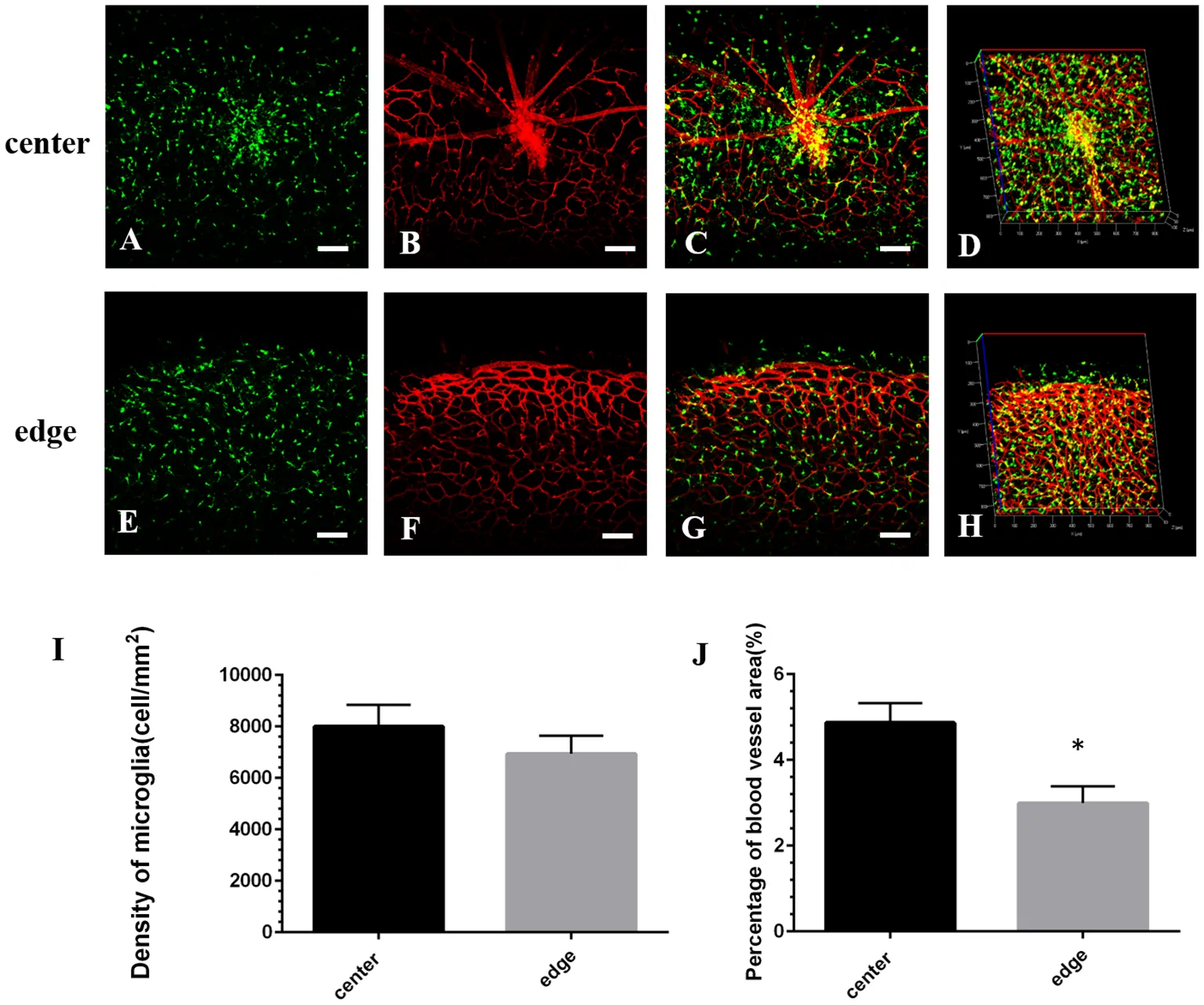
Microglia and vessels at P10. **(A–H)** Microglia and vessels could be both observed in the center and edge retina at P10. **(I)** The density of microglia in the center and edge retina. **(J)** The percentage of blood vessel area in the center and edge retina. (Statistical differences were processed with the T-test, with a p value of <0.05 for differences between groups, n=8). Asterisks indicate p<0.05.

At P30, microglia and vessels were detected in both the central and peripheral retina (**Figures 6A–H**). The microglia density in the central retina was similar to that in the edge retina (6418.88 ± 1150.33 cells/mm^2^ vs. 6145.45 ± 534.57 cells/mm^2^; p>0.05; n=8) (**Figure 6I**). The percentage of blood vessel area in the central retina (3.19 ± 1.18%) was higher than that in the edge retina (2.18 ± 0.41%); however, the difference was not significant (p>0.05; n=8) (**Figure 6J**).

**Figure 6:**
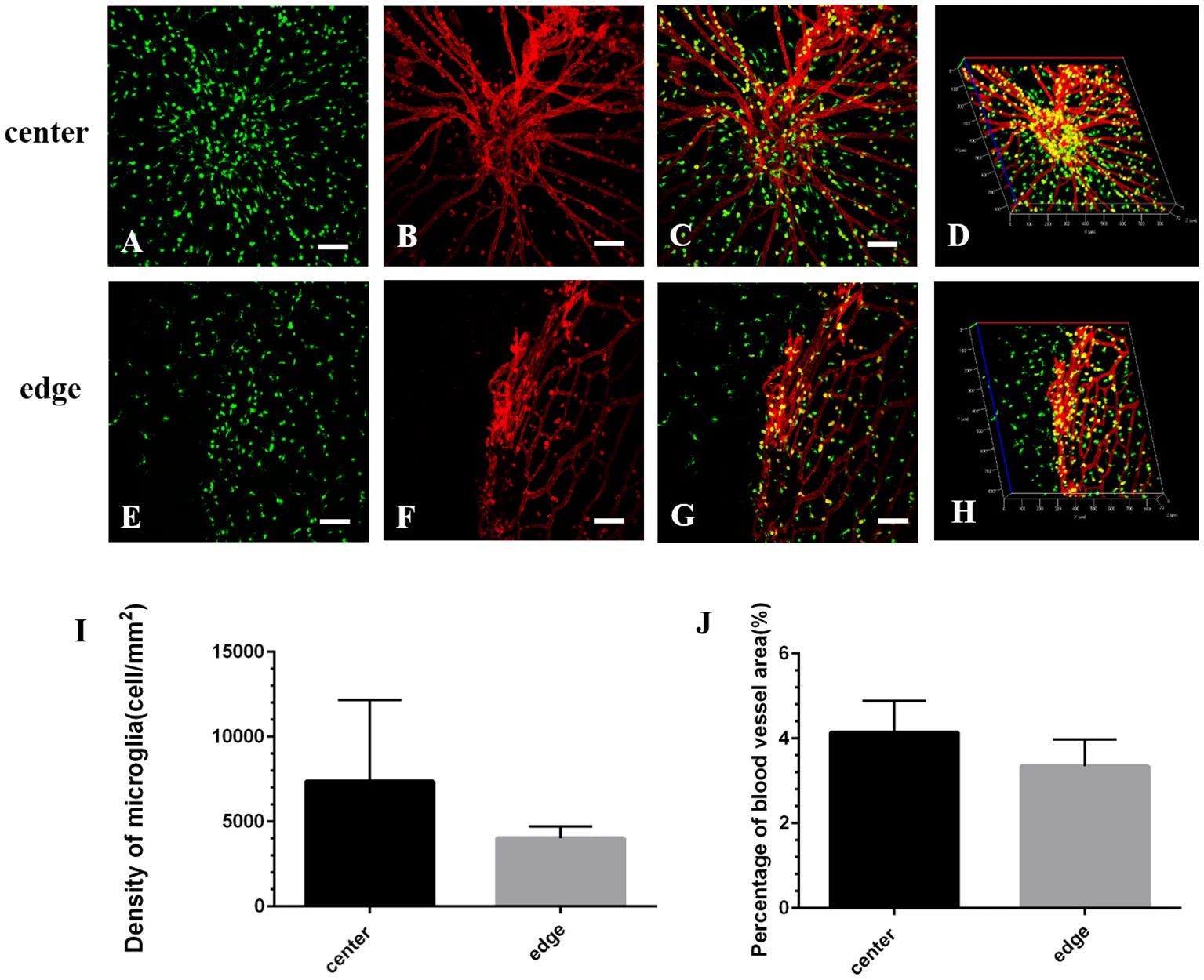
Microglia and vessels at P30. **(A–H)** Microglia and vessels could be both observed in the center and edge retina at P30. **(I)** The density of microglia in the center and edge retina. **(J)** The percentage of blood vessel area in the center and edge retina. (Statistical differences were processed with the Wilcoxon test*, p* < 0.05, for differences between groups, n=8).

The images were enlarged to show the relationship between microglia and vessels during retinal development. At P1, microglia were observed in the outermost periphery of the retina; however, vessels were not detected in these regions (**Figure 7A**). From P10 to P30, microglia and vessels were observed in the outermost periphery of the retina (**Figures 7B, C**). At P1, microglia had a larger cell body, which was round or oval, and emitted only thick and short protrusions from the cell body (**Figure 7D**). At P10, microglial cells were spindle-shaped, with two or more protrusions located at both ends of the cell body. Furthermore, the cells were aligned parallel to the nerve fiber direction (**Figure 7E**). At P30, the microglial cell body was relatively round and small, with multiple processes around the cell body and secondary processes (**Figure 7F**).

**Figure 7:**
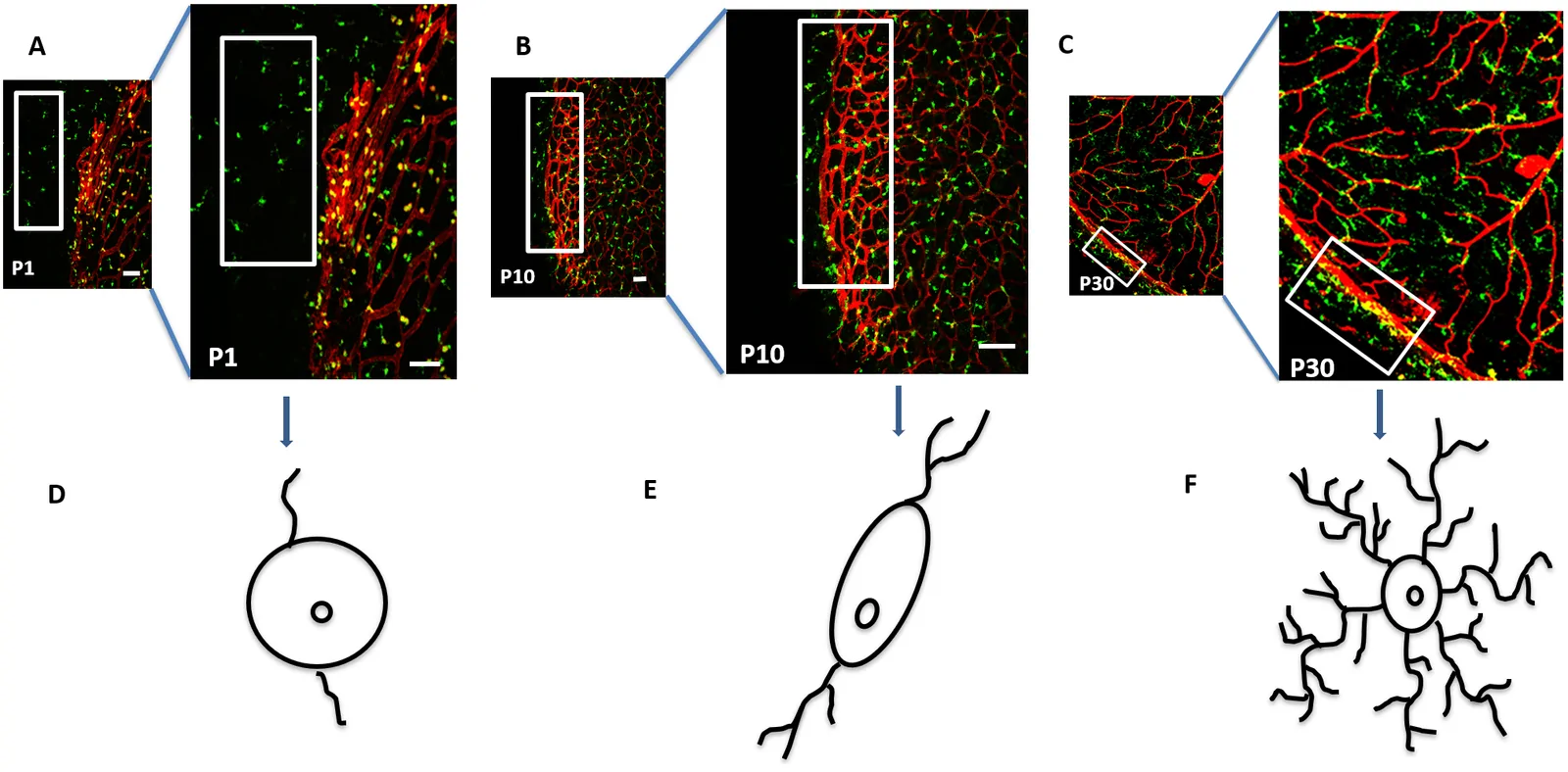
The developmental sequence between microglia and vessels at P1, P10, and P30. **(A)** Microglia could be observed in the outermost periphery of the retina. However, vessels were not observed at P1. **(B–C)** From P10 to P30, both microglia and vessels were observed in the outermost periphery of the retina. **(D)** At P1, microglia had a larger cell body, which was round or oval. It emitted only a thick and short protrusion from the cell body. **(E)** At P10, the microglial cell was spindle-shaped, with two or more protrusions located at both ends of the cell body. The cells were always aligned parallel along the direction of the nerve fiber. **(F)** At P30, the microglia cell body was relatively round and small, with multiple processes around the cell body and secondary processes (Image 7F).

Minocycline (33 mg/kg) was intraperitoneally injected once daily. The results showed that microglia in the retina and vessels were significantly reduced in the minocycline group compared with the normal group at P1 and P10 (**Figure 8A**). The percentage of microglia away from the vessels was quantified using the StrataQuest software (version 7.0.1). At P1, the percentage of microglia (0–1.7 µm away from vessels) in the minocycline group was significantly lower than that in the normal group (51.6 ± 1.7% vs. 81.51 ± 4.3%), and the difference was significant (p<0.05; n=8). At P10, the percentage of microglia (0–1.7µm away from vessels) in the minocycline group was higher than that in the normal group (81.34.34 ± 4.7% vs. 43.51 ± 5.1%), and the difference was significant (p<0.05; n=8). At P1, the percentage of microglia (1.7–20 µm away from vessels) in the minocycline group was significantly higher than that in the normal group (18.14 ± 0.87% vs. 6.89 ± 2.13%), and the difference was significant (p<0.05; n=8). At P10, the percentage of microglia (1.7–20 µm away from vessels) in the minocycline group was higher than that in the normal group (18.47 ± 3.26% vs. 15.38 ± 2.65%); however, the difference was not significant (p>0.05; n=8). (**Figure 8B**).

**Figure 8:**
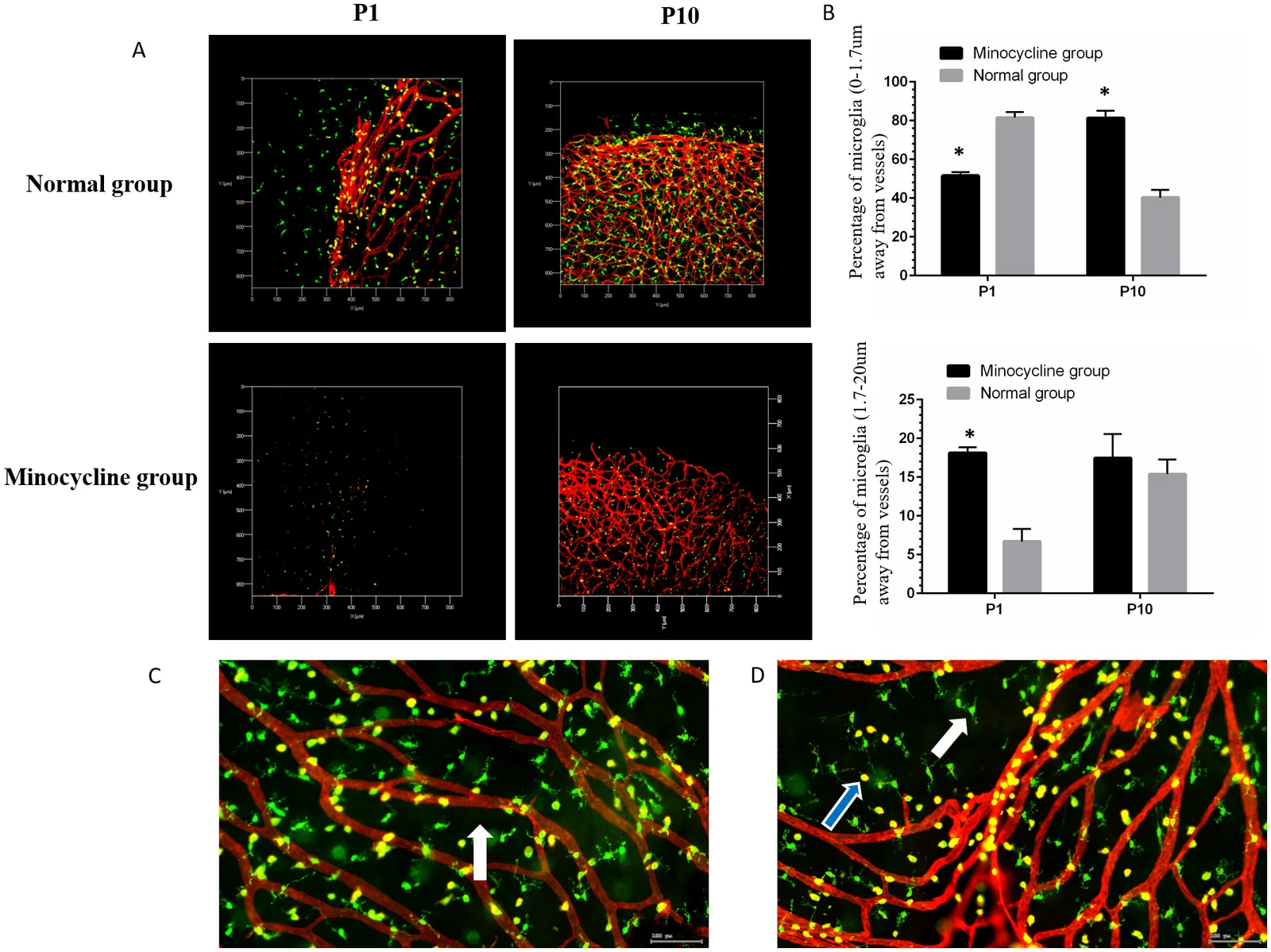
Microglia and vessels in the minocycline group and normal group at P1 and P10. **(A)** Minocycline was injected intraperitoneally once a day. Microglia and vessels in the retina were significantly reduced in the minocycline group at P1 and P10. **(B)** The percentage of microglia away from vessels was quantified using StrataQuest software. **(C, D)** Microglia cell bodies and vessels were identified. (Statistical differences were processed with the Wilcoxon signed-rank test, with a p value of <0.05 for differences between groups, n=8).

Importantly, we used StrataQuest software (version 7.0.1) to identify microglia cell bodies and vessels, including the recognition of vessels (the white arrow indicates the vessel) (**Figure 8C**) and the recognition of microglia cell bodies (the yellow cells in this image are indicated by the blue arrow as the cell bodies that activate the microglia, and the white arrows point at the resting microglia) (**Figure 8D**).

## Discussion

Owing to its easy accessibility, the mouse retinal vasculature is an excellent model for studying vascular growth and remodeling during the development process or under pathological conditions. Progress in imaging techniques and analysis methodologies would facilitate comprehension of the retinal vasculature. Our findings agree well with previous studies that have shown that the superficial plexus of the mouse retina is well-developed during the first week after birth. Subsequently, angiogenic sprouts from the superficial layer grow into the retina during the second week to form a deep layer (Stahl et al., 2010). Similar studies have also shown that the mouse retinal vasculature spreads radially from the optic nerve head peripherally toward the ganglion cell layer during the first postnatal week, and the development of the vascular network completes by the third postnatal week (Yagasaki et al., 2014). In this study, we found that retinal vessels start from the center of the visual papilla and radiate to the peripheral retina. Vascular remodeling began at P7. Subsequently, the vascular network formed a honeycomb-like structure. The arterial and venous arteries could be differentiated by their diameter at P14. At P17, vascular remodeling occurred, and a complete vascular network was formed. We confirmed that the length of the great vessels increased gradually while the number of primary branches decreased. We characterized the gradual growth of the retinal vasculature and the dynamic remodeling processes. This set of data can serve as a basis for the genetic or pharmacological manipulation of the retinal vasculature.

Furthermore, we determined whether the developmental process of vessels is affected by microglia. We found that the appearance of microglia precedes the appearance of vessels at P1. Minocycline (33 mg/kg) was intraperitoneally injected once daily (Kobayashi et al., 2013). Compared to the normal group, microglia and vessels in the retina were significantly reduced in the minocycline group at P1 and P10, which could be seen by the naked eye. The analysis of the percentage of microglia and vascular distance found that the percentage of microglia (0–1.7 µm away from blood vessels) in the minocycline group was lower than that in the normal group at P1, and the difference was significant. These results indicate that minocycline inhibited the activation of microglia and thus affected the degree of the close connection between microglia and blood vessels at P1.

The retina and its clinical disorders play a pivotal role in angiogenesis research and provide model systems for investigating neurovascular relationships and angiogenic diseases. Abnormal vascular development plays a vital role in the occurrence and development of retinal diseases (Carmeliet and Tessier-Lavigne, 2005). The development of retinal vessels is often affected by factors such as diabetes, trauma, aging, dyslipidemia, genetic predisposition, and environmental stress. These injurious stimuli commonly arrest vascular development or cause vascular occlusion. They can cause the retina to lose vessels and oxygen supply. In turn, excessive production of growth factors, such as VEGF, is stimulated, leading to the disorder of intravitreal angiogenesis. New vessels in the vitreous cavity can cause retinal detachment through the contraction of fibrous vessels under the retina. The subsequent pathological response generates disorganized, leaky, and tortuous vessels that leak into the interface between the vitreous and retinal tissues, attracting fibroglial elements that cause severe hemorrhage, retinal detachment, and vision loss (Yan et al., 2015).

Microglial cells, which are phagocytic dendritic cells with immune functions, co-distribute with retinal vessels during development and are activated in ischemic diseases. However, their role in retinal vascular information, if any, remains unknown. This study showed that microglia develop in the retina prior to vessel growth. Microglia play a significant role in physiological and pathological retinal angiogenesis. These results further demonstrate the critical role of microglial activation in driving subretinal neovascularization (Usui-Ouchi et al., 2020). In recent studies, retinal microglia have been shown to influence pathological neovascularization, likely through the exchange of cellular signals with associated vascular elements (Zhao et al., 2009). Microglial cells are intimately associated with retinal vessels during vasculogenesis early in life, during normal development, and during vascular repair and neovascularization under pathological conditions (Checchin et al., 2006; Ritter et al., 2006). In models of ischemic retinopathy, retinal microglia cell density is dramatically increased. When these cells are artificially depleted, increased vascular pathology ensues (Davies et al., 2006). Therefore, microglia are involved in mediating the vascular remodeling of the retina. This study showed that microglia develop in the peripheral retina before vessel growth.

Overall, these results indicated that the emergence of microglia takes priority over the formation of retinal vessels. Retinal microglia comprise a heterogeneous population of cells that are believed to be of hemangioblastic mesodermal origin (Streit, 2001). During human retinal development, microglia lacking macrophage markers are present at 10 weeks of gestation (WG), before astrocyte invasion and the onset of vasculogenesis. In contrast, those positive for macrophage antigen (S22) appear at approximately 14 WG, the beginning of vascularization (Diaz-Araya et al., 1995). It has been speculated that microglia extend their processes to orient toward and contact vessels as they form (Pennell and Streit, 1997). Therefore, it is reasonable to assume that microglia play an important fundamental and preparatory role in retinal vascular development. Moreover, it has been shown that another type of glial cell called astrocyte also directs the growth of vessels; these cells are made up of pre-existing astrocytes (Gariano and Gardner, 2005). If microglia serve as a template for growing vessels, the factor guiding them through the process needs to be identified. One factor might be the pre-existing radially orientated axon bundles of ganglion cells, which are retinal output neurons that project through the optic nerve to the brain’s visual centers. Microglial growth roughly follows this radial plan in a process that requires platelet-derived growth factors synthesized by ganglion cells (Gariano and Gardner, 2005). Vessels and glia often use attractive and repulsive guidance cues from axons (Carmeliet and Tessier-Lavigne, 2005).

Cell biologists are now capitalizing on the accessibility of the retina to investigate the important aspects of developmental angiogenesis, including how it relates to neuronal and glial development, morphogenesis, oxygen sensing, and progenitor cells. Since retinal vascularization occurs in a timely, highly coordinated fashion and can vary considerably between mouse strains, it must be noted that the presented data only apply to retinal vascular development in CX3CR1 mice. Therefore, further studies using different animal models must confirm this conclusion.

## Data Availability

The original contributions presented in the study are included in the article/supplementary material. Further inquiries can be directed to the corresponding author.
